# Sex Differences Influence ERP Components in Semantic Decisions

**DOI:** 10.3390/brainsci16070713

**Published:** 2026-07-02

**Authors:** Fabiola R. Gómez-Velázquez, Vanessa D. Ruiz-Stovel, Carlos González-Medina, Sergio A. Nava, Aurora Espinoza-Valdez

**Affiliations:** 1 Instituto de Neurociencias, CUCBA, Universidad de Guadalajara, Guadalajara 44130, Mexico; vanessa.ruizstovel@academicos.udg.mx (V.D.R.-S.); carlos.gonzalez9342@alumnos.udg.mx (C.G.-M.); sergionavro@gmail.com (S.A.N.); 2 Departamento de Ciencias Computacionales, CUCEI, Universidad de Guadalajara, Guadalajara 44430, Mexico; aurora.espinoza@academicos.udg.mx

**Keywords:** semantic decision, ERPs, sex differences, N400, P300

## Abstract

**Highlights:**

**What are the main findings?**

- Sex differences in semantic sentence processing emerged during both anticipatory and integrative stages, with females showing larger N400/N2 responses and males showing larger P300 amplitudes;
- ERP differences appeared before the sentence-final word, supporting predictive models in which semantic expectations are progressively built during reading.

**What are the implications of the main findings?**

- Semantic prediction during reading is a dynamic, stage-dependent process affected by sex-related neurocognitive differences;
- Combining males and females in grand-averaged ERP analyses may mask meaningful neurophysiological differences, underscoring the importance of accounting for sex as a biological variable in language research.

**Abstract:**

**Background/Objectives:** Prediction is a fundamental mechanism of language comprehension, enabling readers to generate expectations about upcoming words during sentence processing. Event-related potentials (ERPs), particularly the N400 and P300, provide sensitive electrophysiological markers of semantic integration and expectancy-related processing. However, the contributions of individual sentence elements to semantic prediction and the influence of sex differences on these processes remain poorly understood. **Methods:** Forty-six healthy right-handed young adults (23 females) performed a semantic decision task with six-word Spanish sentences ending in congruent or incongruent final words while EEG activity was recorded. ERP components associated with anticipatory and integrative stages of sentence processing were analyzed during the sequential presentation of sentence words. Behavioral performance and sex-related ERP differences were assessed using repeated-measures ANOVAs and correlational analyses. **Results:** Behavioral findings showed that males committed significantly more errors in detecting semantic incongruities, although overall performance exceeded 92%. Congruent sentence endings elicited a centro-parietal P300, whereas incongruent endings produced a robust N400 followed by a late positive component (LPC). Sex differences emerged, beginning at the third word of the sentence. Males exhibited greater P2 amplitudes and a larger anticipatory P300-like positivity preceding the final word, whereas females showed enhanced N2 and N400 negativities associated with contextual processing and semantic incongruity. Correlations between anticipatory ERP activity and later semantic components supported functional continuity between predictive and integrative stages of language processing. Despite earlier differences, males and females exhibited comparable LPC amplitudes, suggesting convergence during later elaborative processing stages. **Conclusions:** These findings support predictive models of language comprehension by demonstrating that semantic expectations are progressively constructed throughout sentence processing. Sex-related ERP differences were observed across anticipatory, attentional, and semantic integration stages, indicating quantitative and stage-specific neurophysiological variations rather than qualitatively distinct language-processing strategies.

## 1. Introduction

It is widely accepted that prediction is a crucial feature of language comprehension (see [1] for a review). Because sentences unfold linearly over time, we use information from preceding words to generate expectations about upcoming words. Based on prior experience, top-down mechanisms pre-activate the most likely upcoming words, thereby facilitating and accelerating their linguistic processing [1,2,3].

However, several authors question the viability of linguistic prediction because the same sentence can be constructed in multiple ways (e.g., [4,5,6]). This perspective holds that comprehension proceeds in a stimulus-driven, constrained manner. In an ERP study using spoken sentences, Leon-Cabrera et al. [7] compared highly constraining sentences (e.g., “a good magician never reveals his … tricks”) with low-constraining ones (e.g., “they agreed to meet the next … morning”). They found a frontal slow-negative potential shift during the anticipatory period preceding the final word, with greater amplitude in highly constraining sentences. This was interpreted as a probable index of specific semantic predictions, consistent with the interpretation proposed by Grisoni et al. [8].

More recently, León-Cabrera et al. [9] applied a similar methodology to evaluate linguistic prediction, presenting sentences visually, word by word, with varying degrees of semantic constraint. They observed a slow left negativity peaking in the interval preceding the closing word, which they interpreted as an index of engagement of cognitive operations associated with semantic prediction. These findings confirm two central notions: (1) sentence closure might be progressively built from the sequentially incoming information provided by the initial constituent words, and (2) ERP tools seem to be effective in evaluating the construction process of linguistic prediction in sentences.

One central, still open question is the relative contribution of each constituent word in a sentence to the semantic prediction of its closing element. More precisely, is it possible to identify an electrophysiological brain marker of this prediction earlier in the sentence’s construction process?

Several ERP components have been extensively studied during word and sentence reading, highlighting the left-lateralized N170, which reflects the specialized neural processes that underlie visual expertise for print across different writing systems [10,11,12]; P200, a fronto-centrally distributed ERP component modulated by access to the orthographic word-form, associated with early stages of lexical processing and representing an index of holistic word-form recognition [13,14,15,16,17]; and P300, particularly the subcomponent P3b, which has been argued to arise when attentionally mediated processes promote memory operations in temporo-parietal areas [18]. In this regard, P3b might reflect the results of decision-making processes [19,20].

The N400 is probably the best-known brain index of language understanding. It is strongly affected by expectations, and its amplitude depends on how predictable a word is within its sentence context [1,21,22]. The role of the N400 as a reflection of prediction errors and its neural basis has been widely discussed in recent years (see [23,24,25] for extensive reviews and discussion). Finally, a post-N400 late parietal positivity component (LPC) involved in language comprehension experiments (usually described as P600, typically observed between 500 and 900 ms after the onset of a critical word or phrase) has been shown to be sensitive to syntactic ambiguities and violations (e.g., [26,27,28]). It is usually interpreted as a marker of structural processing, such as re-analyses and efforts to build a coherent sentence structure [27,28,29]. More recently, LPC has been found to predict later recognition memory at both the behavioral and neural levels [30]. This LPC has also been interpreted as possibly capturing a domain-general process that likely precedes subsequent operations involved in understanding the meaning of a sentence, including recovery from a violation or reanalysis of a sentence’s contents [31].

Spanish has far more flexibility in word order than English. However, both languages can produce similar sentence structures in which the final word affects the overall meaning of the sentence. Building on the varied roles each sentence element plays in shaping possible semantic closures and the proven value of ERP tools for assessment, this study focuses on a key question highlighted in the previous literature on sex-related differences in language processing: are ERP differences detectable across constituent words of a sentence, and are these differences consistent across sexes?

There are well-documented sex-related differences in brain function during word processing from early neurodevelopmental stages onward. Indeed, language is considered a lateralized cerebral function, and this lateralization is essential for the full development of linguistic abilities [32,33]. The seminal study by Shaywitz and colleagues [34] demonstrated significant sex-related differences in brain activation patterns during phonological tasks. Males showed strong lateralization to the left inferior frontal gyrus, whereas females engaged more diffuse neural systems involving both the left and right inferior frontal gyri.

Furthermore, several studies have demonstrated that females perform better on reading tasks and are less susceptible to dyslexia, regardless of their language or the educational system they are exposed to (see [35] for a recent review). Some authors have proposed that processing speed, along with inhibition and verbal reasoning, is a critical factor in explaining sex differences in reading performance, while disregarding phonemic awareness and working memory as intervening causes [36]. Moreover, since primary school, girls have been able to spell significantly better than boys [37]. Women are also significantly faster than men in speech-based coding, and they have an advantage in both prelexical and lexical processing, which probably leads to a sex difference in the quality of their phonological representations [38].

Regardless of sex, reading represents a crucial acquisition for any individual to develop integrally. Reading relies on visual word recognition, which involves short- and long-term memory mechanisms that represent coherent information and determine comprehension. The linguistic process that enables semantic prediction is undeniably linked to more complex reading skills. However, the specific contribution of each word element in a sentence to predicting its closure remains far from established, particularly when explicitly considering potential distinctions based on the reader’s sex and the brain’s underlying functional substrates. This study aims to evaluate, using ERP tools, the sex differences in the predictive process of sentence closure, assessing the electrophysiological changes that occur at each step of the process -the presentation of each sentence’s constituent word- in a shallow language such as Spanish.

## 2. Materials and Methods

**Participants.** Undergraduate students were recruited from both private and public universities in Guadalajara, Mexico. A total of 50 students agreed to voluntarily participate in the study; however, the final study sample was composed of 46 right-handed young adults (23 males) since three male participants were excluded due to excessive EEG artifacts, and another male participant was excluded due to early protocol abandonment. The undergraduate students were selected from three distinct majors to account for the potential influence of academic discipline on reading experience: Medicine (7 males and 5 females), Psychology (8 males and 14 females), and Engineering (8 males and 4 females). The sample’s mean age was 21.5 years (SD = 1.5), reading speed was 170.7 words per minute (SD = 21.1), reading errors were 2.0 (SD = 1.3), reading comprehension was 72% (SD = 20.3%), and estimated IQ (using the Vocabulary and Block Design subtests from WAIS IV) was 112.5 (SD = 9.3). They had no personal history of psychiatric or neurological conditions. Table 1 presents the demographic characteristics of the sample. All participants’ reading performance was within normal parameters according to the reading speed norms published by the Mexican Ministry of Education.

**Figure 1:**
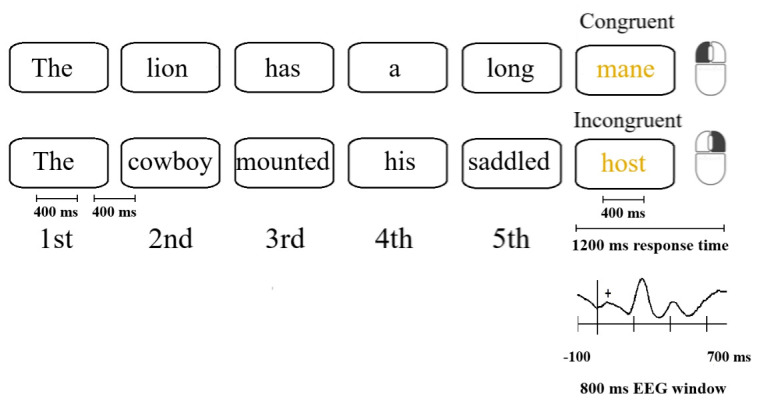
Schematic representation of the semantic decision task. The words were presented one by one, from the first to the sixth. The sixth word was displayed in yellow. The analysis window used to obtain the closing word ERPs is represented at the bottom of the figure.

**Procedure.** We recorded electrical brain activity in an acoustically and electrically shielded room during the performance of a semantic decision task, adapted from [39], consisting of 68 six-word sentences presented in pseudorandom order. At the end of every sentence, the participant indicated whether the last word was congruous (34 trials) or incongruous (34 trials) with the rest of the sentence (see Figure 1). Each word was presented on a 19-inch screen in white font on a black background for 400 ms, with an interstimulus interval of 400 ms and an intertrial interval of 800 ms. Only the sixth word was presented in yellow to indicate that it was the last word in the sentence. Participants were instructed to respond as accurately and quickly as possible using their right hand to press either the left or right mouse click button. They had a total of 1200 ms to indicate whether the closing word was congruous or not. Stimulus presentation and response collection were controlled by the Mindtracer software 2.0 (Neuronic, S.A., Havana, Cuba).

**Recording.** EEG activity was recorded from scalp sites: Fp1, Fp2, F3, F4, F7, F8, C3, C4, P3, P4, O1, O2, T3, T4, T5, T6, Fz, Cz, and Pz, according to the 10–20 international system, using a commercial electrode cap (Electro-Cap International, Inc., Eaton, OH, USA). Linked mastoids served as reference electrodes, and an EOG recording was obtained from the outer canthus and a frontal placement. The EEG and EOG signals were amplified with a 0.5–30 Hz band-pass filter (including a 60 Hz notch filter), sampled at 4 ms (250 Hz), on the MEDICID-05 (Neuronic, S.A., Havana, Cuba). Electrode impedance was kept below 5 kΩ.

**ERP averaging**. The initial time instant (t0) corresponded to the presentation of each word in the sentence trial. The EEG epochs were 800 ms in length, with the ERP time window defined as 100 ms before stimulus onset to 700 ms after stimulus onset. The pre-stimulus window was used for baseline correction. For all participants, epochs of data on all channels were excluded from averages when the voltage in a given recording epoch exceeded 100 µV on any EEG or EOG channel. Each individual ERP reached a standard deviation rate (SDR) below 1.1 and a residual noise level (RNL) below 2. Single-trial data were visually inspected for artifact rejection by two experts before individual and group averaging.

For the first five words, at least 40 artifact-free epochs were averaged per participant regardless of the sentence’s closing word condition. Then, grand-mean waveforms for each sex (23 participants per group) were analyzed to obtain the homologs of N170, P200, N200, and P300. For the closing word, a total of 25 ERP artifact-free, correct-response time windows per condition (congruous/incongruous) per participant were averaged. Group averages were also analyzed to evaluate the P300, N400, and P600 components associated with the semantic decision choice.

All procedures were submitted to and approved by the Neuroscience Institute’s Ethics Committee (ET082022-353, Universidad de Guadalajara) before testing, ensuring that the experiment was conducted in compliance with relevant laws and institutional guidelines and in accordance with the Declaration of Helsinki. All participants agreed to participate voluntarily and gave their written informed consent.

## 3. Results

### 3.1. Behavioral Responses

In the initial reading skills and estimated IQ evaluation, sex differences were observed only in reading errors, with males making more errors than females (Mann–Whitney U = 170.5, *p* < 0.05), see Table 2. No differences were found among participants based on their undergraduate majors.

For the analysis of performance on the semantic decision task, equality of variances was assumed (Levene’s test), and therefore, a one-factor ANOVA (sex) was used. It was found that males made more errors in detecting semantic incongruities (F(1,45) = 5.428, *p* < 0.05, η^2^*_p_* = 0.110), with no differences observed in the other behavioral variables. Overall task performance exceeded a 92% accuracy rate.

#### Correlations

In the behavioral results of the semantic decision task, only a correlation was observed in the incongruent condition across all participants: correct responses were negatively correlated with reaction time (*r* = −0.430, *p* < 0.01).

### 3.2. ERP Grand-Mean Waveforms

**Figure 2:**
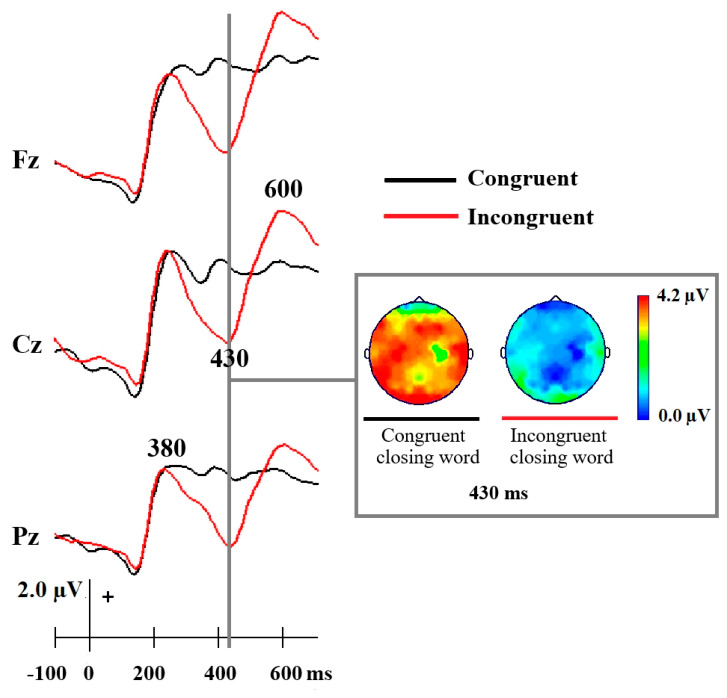
Semantic decision task grand-mean waveforms. Average ERPs (*n* = 46) in mid-frontoparietal electrodes (Fz, Cz, and Pz) are shown for the congruent condition (black line) and incongruent (red line). The gray box shows the topographic distribution of the maximum negative peak at 430 ms.

Participants of both sexes were included in the first ERP grand-mean waveform analysis to evaluate the classic semantic incongruency effect at the sixth word. Electrophysiological responses to the closing word condition showed a typical midline pattern of N1-P2 waveforms, followed by either a P300 or an N400 component, depending on whether the sentence’s final word was semantically congruent or incongruent, respectively, and a prominent later fronto-central LPC after the N400. Figure 2 shows the midline grand-averaged ERP waveforms for the entire sample (disregarding sex) corresponding to the time window when the sentence closing word was presented. A predominantly parietal positivity peaking around 380 ms corresponded to the congruent closure, while a significant fronto-central negativity peaking at 430 ms corresponded to the detection of the semantically incongruent word.

### 3.3. ERP Sex Differences in the Semantic Decision Task

A second analysis examined sex differences in the ERP amplitudes of the components observed in the closing word conditions: P300, N400 and LPC. The ERP peak amplitudes were compared using a repeated-measures (RM) ANOVA with two within-subject factors, condition (congruent vs. incongruent) and recording site (F3, F4, C3, C4, P3, P4, Fz, Cz, Pz), and one between-subject factor, sex (men vs. women). Individual maximum voltages were measured within +/− 50 ms around each group’s peak average and each component was analyzed independently. Additionally, post hoc tests were calculated to explore any trends in the observed differences using Bonferroni adjustments for multiple comparisons.

- Condition main effect: expected differences were observed in the semantic decision task, with a very large effect size (F(1,44) = 108.758, *p* < 0.001, η^2^*_p_* = 0.712). A congruent closing word elicited a P300 around 405 ms. In contrast, a semantically incongruent closing word elicited the typical N400 component at 416 ms.
- Recording site effect: a significant main effect was found for P300 amplitude (F(8,352) = 8.782, *p* < 0.001, η^2^*_p_* = 0.166) across the nine central electrode sites. Bonferroni post hoc comparisons revealed that peak amplitudes at the left parietal electrode site (P3) were significantly greater than those observed at the three frontal sites (*p* < 0.01) and the remaining two parietal sites (*p* < 0.01).
- The effect of Sex: males showed greater overall positivity than females (F(1,44) = 11.644, *p* < 0.01, η^2^*_p_* = 0.209). No Condition × Sex interaction was found, likely because the differences were in the same direction.

An independent analysis of the Sentence-Final Word in each Condition was conducted using a RM-ANOVA with one within-subject factor, recording site (F3, F4, C3, C4, P3, P4, Fz, Cz, Pz), and one between-subject factor, sex (men vs. women).

**Figure 3:**
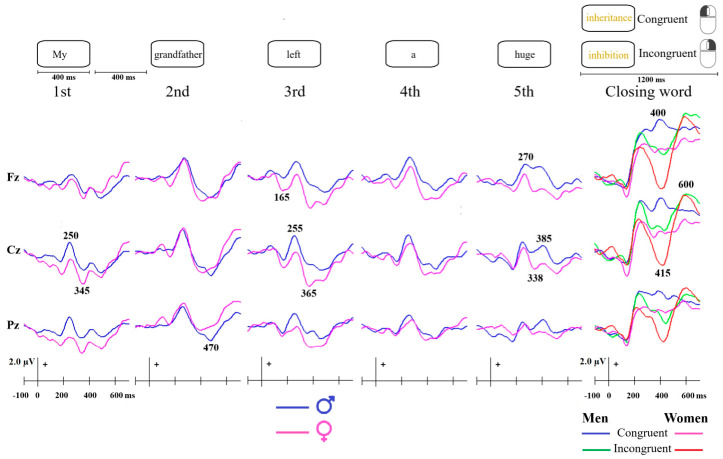
Average ERPs components for each word in the sentence. The first five words display the average ERPs for men (blue) and women (pink). The sixth word is the closing word, and the waveforms for the congruent (men: blue; women: pink) and incongruent (men: green; women: red) conditions are shown.

- In the Congruent condition, males showed a significantly larger P300 component than females (F(1,44) = 6.657, *p* < 0.05, η^2^*_p_* = 0.131); in the Incongruent condition, females showed a significantly larger (more negative) N400 component than males (F(1,44) = 11.837, *p* < 0.01, η^2^*_p_* = 0.212).
- The presence of an incongruent word also elicited a late positive component (LPC) that was significantly larger than that for the congruent word (F(1,44) = 26.057, *p* < 0.001, η^2^*_p_* = 0.372). For this component (Figure 3), no differences were observed between men and women (F(1,44) = 0.687, *p* = 0.412, η^2^*_p_* = 0.015).

### 3.4. Sex Comparison Across Words and Components

A third analysis focused on the expectancy context created by the preceding words in the sentences (Figure 3). Sex differences were examined in the first five words of each sentence across five ERP components, initially denoted as P1, N1, P2, N2, and P300-like based on their polarity. Figure 3 shows the grand-averaged ERP waveforms for each sex during the presentation of each sentence’s constituent word. The maximum voltage was measured within a 100 ms window around each group’s peak average (+/- 50 ms), and the locations where they reached their maximum amplitude or predominantly occurred were further analyzed: P1 and N1, only T5 and T6 sites were included; P2 and N2: fronto-central predominance (F3, F4, C3, C4, Fz, Cz).; N2 (posterior distribution): centro-parietal predominance (C3, C4, P3, P4, Cz, Pz).

An RM-ANOVA was conducted independently for each component with word (5) and recording site (2 or 6) as within-subject factors, and sex (2) as a between-subject factor. Bonferroni adjustments for multiple comparisons were explored as needed.

- No sex differences were observed in Words 1 and 2.
- Word 3: Sex differences emerged at Word 3. The P2 generated greater positivity in males (F(1,44) = 5.259, *p* < 0.05, η^2^*_p_* = 0.107), and N2 greater negativity in females (F(1,44) = 9.176, *p* < 0.01, η^2^*_p_* = 0.173).
- Word 4: Sex differences appeared in N2 with greater negativity in females (F(1,44) = 4.087, *p* < 0.05, η^2^*_p_* = 0.085).
- Word 5: Similar sex differences were observed in N2 (greater negativity in females; F(1,44) = 5.044, *p* < 0.05, η^2^*_p_* = 0.103). In Word 5 (preceding the sentence-final word), a P300-like positivity (~400 ms) with greater amplitude in males was observed (F(1,44) = 11.837, *p* < 0.01, η^2^*_p_* = 0.212). This component appears to anticipate the final word but was prominent only in males.

### 3.5. General Differences Between Words (Word × Site: F3, F4, C3, C4, Fz, Cz) per Component

- P2 Component: There were significant differences in the amplitude of this component across words (F(4,180) = 7.946, *p* < 0.001, η^2^*_p_* = 0.150). Word 2 showed greater P2 positivity than the others (it is the first word with semantic content—mostly nouns—unlike Word 1, which consisted of articles or pronouns), and there was a significant word x site interaction (F(20,900) = 4.861, *p* < 0.001, η^2^*_p_* = 0.091). The increase in Word 2 was greater at frontal than at central sites. Post hoc analyses indicated that this regional difference was statistically significant (*p* < 0.01).
- N2 Component: There were also significant differences among words (F(4,180) = 4.351, *p* < 0.01, η^2^*_p_* = 0.088). Amplitude significantly decreased in Word 5, particularly at Cz (*p* < 0.01).
- P300-like component (C3, C4, P3, P4, Cz, Pz): Significant differences between words were found (F(4,180) = 11.327, *p* < 0.001, η^2^*_p_* = 0.201). This component was observed only in Word 5, showing a significant increase in positivity compared with when Words 2, 3, and 4 (*p* < 0.01) were presented. The P300 electrode site effect (F(5,225) = 32.572, *p* < 0.001, η^2^*_p_* = 0.420) was significantly larger at parietal sites than at central sites (*p* < 0.01).

### 3.6. Correlations

Pearson correlations for the electrophysiological data were estimated across the 46 participants. The P300 peak maximum amplitude in Word 5 significantly correlated with P300 peak maximum amplitude in the closing word congruent condition at Fz (*r* = 0.399), Cz (*r* = 0.456), and Pz (*r* = 0.484), all correlations with a significance level of *p* < 0.01. Additionally, the N2 peak maximum amplitude in Word 5 showed a significant correlation with the N400 peak maximum amplitude in the closing word incongruent condition at Fz (*r* = 0.429), Cz (*r* = 0.420), and Pz (*r* = 0.490) all with a significance level of *p* < 0.01 at each electrode site.

### 3.7. Simple Discriminant Analysis:

A discriminant analysis was conducted to classify males and females using mean ERP amplitudes from the six recording sites, with each condition analyzed separately. The assumption of equal covariance matrices was met in both cases.

- Congruent condition (P300): Overall accuracy rate for predicted group membership was 76.1% (Wilks’ λ = 0.634, χ^2^ = 18.028 (df 9), *p* < 0.05), with 73.9% for men and 78.3% for women;
- Incongruent condition (N400): Overall accuracy rate for predicted group membership was 73.9% (Wilks’ λ = 0.676, χ^2^ = 15.672 (df 8), *p* < 0.05), with 65.2% for men and 82.6% for women.

In general, after task performance, a widely distributed P300 component was observed in the Congruent condition, and N400 and LPC were observed in the Incongruent condition. Furthermore, a left-lateralized N170 component was observed for all words, and a perceptual decoding P200-like frontal component (see Figure 3) was observed for the first 5 words, with greater amplitude in the second word, which could be interpreted as a predictive index of semantic closure.

## 4. Discussion

This study examined behavioral performance and event-related potentials (ERPs) during a classic semantic decision task in young adults, with particular emphasis on sex differences across sentence processing stages. Three principal findings emerged. First, at the behavioral level, men made more errors than women in detecting semantic incongruities, although overall performance was high (accuracy > 92%). Second, at the electrophysiological level, the expected dissociation between the P300 to congruent sentence-final words and the N400 to incongruent endings was robust, with a very large condition effect. Third, systematic sex differences were observed in both early–mid latency components (P2/N2) and late components (P300/N400), including anticipatory activity preceding the sentence-final word. As a final but interesting result, a late parietal positivity showed similar amplitudes in males and females.

### 4.1. Behavioral Performance and Semantic Monitoring

Although overall accuracy was high, men made significantly more errors than women in detecting semantic anomalies. This pattern aligns with evidence of subtle female advantages in language-related tasks, particularly those requiring verbal fluency, semantic retrieval, or fine-grained linguistic monitoring [40,41]. Meta-analytic and neuropsychological data indicate that women often show small but reliable advantages in verbal accuracy, whereas men sometimes show faster response tendencies at the expense of accuracy [42], as reflected in the behavioral responses of this study.

Importantly, the negative correlation between correct responses and reaction time in the incongruent condition suggests that better semantic discrimination was associated with faster anomaly detection. Such findings are consistent with interactive models of language comprehension in which the efficiency of semantic integration reduces decision latency [1].

### 4.2. Canonical N400 and P300 Effects

The main effect of the condition revealed a clear N400 to semantically incongruent endings and a P300 to congruent endings. The N400, peaking around 400 ms, is a well-established marker of difficulty in semantic integration and contextual mismatch [1,21]. Its amplitude is reliably larger (more negative) for semantically incongruent words, reflecting increased integration demands or reduced expectancy [43]. The present findings replicate this canonical effect with a very large effect size, reinforcing the robustness of the paradigm.

Conversely, congruent endings elicited a centro-parietal P300. Although the P300 is traditionally associated with target detection and context updating in oddball paradigms [18], it has also been linked to expectancy confirmation and stimulus evaluation in language tasks [44]. In highly constraining sentence contexts, predictable congruent endings may elicit a P3b-like positivity reflecting context updating and closure [45]. The observed parietal predominance is consistent with the typical scalp distribution of the P3b component [18].

### 4.3. Early and Mid-Latency Differences: P2 and N2

Despite linguistic prediction being a well-recognized phenomenon, the role of pre-N400 components in this process remains a highly debated issue in the literature, with two main supporting hypotheses: (1) Early perceptual processes in the primary sensory cortex facilitate the prediction of a specific word form (the sensory hypothesis, e.g., [46,47], likely involving other brain areas such as the left inferior frontal gyrus and the middle temporal gyrus [48]; and (2) Prediction facilitates the recognition of a word (the word recognition hypothesis, [49,50], which distinguishes word recognition from the semantic integration of word meaning with sentence context and from being merely a reflection of primary sensory cortices’ active processing (see [51], for a comprehensive review).

In the present study, a significant finding was the correlation between N2 and the N400 in the incongruent condition at midline, as well as sex differences in the P2 and N2 components as early as the third word of the sentence. The P2 was larger in men, which might reflect stronger early attentional engagement or a differential early lexical processing strategy [52]. Women, however, exhibited greater N2 negativity from the third word onward, which may index enhanced cognitive control, conflict monitoring, or sensitivity to contextual constraints before sentence closure [53,54]. Such anticipatory N2 effects are consistent with predictive processing frameworks, in which readers continuously generate expectations about upcoming input [55].

The finding that Word 5 (preceding the final word) elicited a P300-like positivity—larger in men—also suggests anticipatory closure processes. As noted earlier, pre-activation of expected lexical items has been shown to modulate ERP components even before critical words appear [45,56]. The correlation between the P300-like response to Word 5 and the P300 to congruent final words further supports the interpretation that this related neural activity changes may together reflect predictive context updating.

### 4.4. Functional Coupling Between Anticipatory and Integrative Processes

Correlations between the P300-like Word 5 and the P300 to congruent endings, as well as between N2 (Word 5) and the N400 to incongruent endings, may indicate there is functional continuity across the anticipatory and integrative stages of processing. This interpretation aligns broadly with predictive coding accounts of language comprehension, in which pre-activation modulates subsequent integration costs [1,55].

The stronger predictive–integrative coupling observed in parietal sites suggests a potential link to the role of posterior cortical networks in context updating and semantic memory integration [57].

### 4.5. Sex Differences in Late Semantic Components

A central finding of this study is the presence of sex differences in both the P300 (congruent condition) and the N400 (incongruent condition). Men exhibited larger P300 amplitudes to congruent sentence-final words, whereas women showed larger (more negative) N400 amplitudes to incongruent sentence endings.

Sex differences in P300 amplitude have been reported across cognitive domains, though findings are mixed and task dependent. Some studies have found larger P3 amplitudes in women, particularly in auditory paradigms [58], whereas others report larger P3b responses in men, depending on stimulus probability and task structure [59]. The larger P300 observed here in men may reflect stronger confirmation of expectations or enhanced context updating when predictions are fulfilled.

In contrast, the enhanced N400 amplitude in women suggests heightened sensitivity to semantic incongruity. Greater N400 amplitudes have been interpreted as reflecting more extensive semantic activation or greater integration effort [1,43]. Prior neuroimaging research indicates that women may recruit language-related cortical networks more bilaterally than men during semantic processing [34,60], potentially supporting more distributed semantic integration. Enhanced N400 responses in women aligns with the possibility of differences in semantic network engagement rather than simple task difficulty.

Notably, the absence of a significant condition-by-sex interaction in the global analysis suggests that these differences reflect amplitude shifts rather than qualitatively distinct processing strategies. However, separate analyses confirmed condition-specific amplitude differences, indicating meaningful sex modulation of late semantic ERPs.

### 4.6. Latest Converging States of Semantic Processing

The presence of a late parietal positivity (LPC) of similar amplitude in both males and females suggests that, despite sex-related differences in earlier processing stages, both groups ultimately engage in comparable levels of elaborative semantic processing. Consistent with the “desirably difficult learning” framework [31], this late positive component has been associated with deeper encoding and integration in language comprehension and signals the re-evaluation of syntactic, semantic, and even orthographic errors [39]. The larger P300 observed in males during congruent trials may indicate a greater reliance on attentional context-updating mechanisms, while the enhanced N400 in females may reflect increased effort in semantic integration. Nevertheless, comparable LPC amplitudes across sexes point to convergence at later processing stages, likely reflecting similar levels of elaborative comprehension and memory encoding [1,45,61].

### 4.7. Discriminant Analysis and Neurocognitive Differentiation

The discriminant analyses yielded classification accuracies of approximately 74–76% based solely on ERP amplitudes, suggesting that sex-related neurophysiological differences are robust enough to support group discrimination. While such classification should be interpreted with caution, it indicates systematic amplitude modulation rather than random variability.

Importantly, these differences should not be construed as categorical or hierarchical but rather as reflecting probabilistic neurocognitive variation within overlapping distributions [62]. The modest effect sizes and high overall task performance underscore substantial commonality in semantic processing across the sexes.

### 4.8. Limitations and Future Directions

The sample consisted of highly educated young adults, limiting generalizability to the young adult mexican population. Hormonal status, menstrual cycle phase, and sociocultural variables were not directly controlled for, even though these factors can modulate ERP amplitudes [63]. Regarding potential hormonal influences, we did not actively control for menstrual cycle phase during testing. However, we collected cycle data to ensure that phases were evenly distributed throughout the female sample, with no single phase being overrepresented. Sociocultural variables were also taken into account during the sample selection process, and hence we included undergraduate students from public and private universities comprising three distinct majors, allowing us to balance between the different academic backgrounds and its potential influence on students’ reading experiences.

Future studies should integrate hormonal measures, source-localization techniques, and connectivity analyses to clarify whether these amplitude differences reflect differences in network-level organization. Longitudinal and developmental approaches would also help determine whether observed sex differences emerge early or are shaped by experience and by educational factors.

## 5. Conclusions

The present findings align with canonical N400 and P300 effects in semantic decision-making and point towards potential systematic sex differences across the anticipatory, attentional, and integrative stages of sentence processing. Women exhibited enhanced N400 and N2 responses to semantic incongruity and contextual processing, whereas men showed larger P300 amplitudes associated with expectancy confirmation and anticipatory closure. Despite the magnitude of earlier ERP sex-related differences, distinct early processing strategies converged at a similar late elaborative processing stage, as suggested by the LPC. These results suggest a pattern compatible with predictive models of language comprehension and may indicate that sex-related differences in semantic processing are quantitative and stage-specific rather than qualitative. Notably, grand-averaged ERP waveforms combining participants of both sexes may inaccurately represent the different stages of sentence reading.

## Figures and Tables

**Table 1:** Demographic and basic neuropsychological data.

	MEN (*n* = 23)	WOMEN (*n* = 23)	*p*
Age	21.3 (1.7)	21.7 (1.3)	0.434
Education (years)	14.1 (0.8)	14.0 (0.7)	0.845
Estimated IQ ^1^	113.7 (9.7)	111.2 (9.0)	0.364
Reading speed (words per minute)	168.7 (22.7)	170.6 (19.9)	0.758
Reading errors	2.3 (1.4)	1.4 (1.0)	0.019
Reading comprehension (%)	73.0 (18.6)	70.4 (22.1)	0.667

Mean (standard deviation); ^1^ Intelligence Quotient.

**Table 2:** Behavioral results in the semantic decision task.

	MEN	WOMEN	*p*
*Congruent*			
Correct responses	31.9 (1.7)	31.1 (2.6)	0.238
Incorrect responses	1.4 (1.4)	1.0 (0.9)	0.174
Reaction time	633.4 (100.7)	656.5 (99.6)	0.439
*Incongruent*			
Correct responses	30.4 (3.3)	30.9 (2.4)	0.582
Incorrect responses	2.3 (2.1)	1.2 (1.0)	0.024
Reaction time	694.9 (83.5)	719.2 (80.4)	0.320

Mean (Standard Deviation).

## Data Availability

The data are not available due to the University of Guadalajara’s policies. They will be available upon request to the first author.
